# In Silico Evaluation, Phylogenetic Analysis, and Structural Modeling of the Class II Hydrophobin Family from Different Fungal Phytopathogens

**DOI:** 10.3390/microorganisms11112632

**Published:** 2023-10-26

**Authors:** Nahla A. Bouqellah, Peter F. Farag

**Affiliations:** 1 Department of Biology, College of Science, Taibah University, P.O. Box 344, Al Madinah Al Munawwarah 42317-8599, Saudi Arabia; 2 Department of Microbiology, Faculty of Science, Ain Shams University, Cairo 11566, Egypt; peter_jireo@sci.asu.edu.eg

**Keywords:** computational annotation, effectors, evolution, homology modeling, hydrophobins

## Abstract

The class II hydrophobin group (HFBII) is an extracellular group of proteins that contain the HFBII domain and eight conserved cysteine residues. These proteins are exclusively secreted by fungi and have multiple functions with a probable role as effectors. In the present study, a total of 45 amino acid sequences of hydrophobin class II proteins from different phytopathogenic fungi were retrieved from the NCBI database. We used the integration of well-designed bioinformatic tools to characterize and predict their physicochemical parameters, novel motifs, 3D structures, multiple sequence alignment (MSA), evolution, and functions as effector proteins through molecular docking. The results revealed new features for these protein members. The ProtParam tool detected the hydrophobicity properties of all proteins except for one hydrophilic protein (KAI3335996.1). Out of 45 proteins, six of them were detected as GPI-anchored proteins by the PredGPI server. Different 3D structure templates with high pTM scores were designed by Multifold v1, AlphaFold2, and trRosetta. Most of the studied proteins were anticipated as apoplastic effectors and matched with the ghyd5 gene of *Fusarium graminearum* as virulence factors. A protein–protein interaction (PPI) analysis unraveled the molecular function of this group as GTP-binding proteins, while a molecular docking analysis detected a chitin-binding effector role. From the MSA analysis, it was observed that the HFBII sequences shared conserved 2 Pro (P) and 2 Gly (G) amino acids besides the known eight conserved cysteine residues. The evolutionary analysis and phylogenetic tree provided evidence of episodic diversifying selection at the branch level using the aBSREL tool. A detailed in silico analysis of this family and the present findings will provide a better understanding of the HFBII characters and evolutionary relationships, which could be very useful in future studies.

## 1. Introduction

Hydrophobins (HFBs) are a family of remarkable surfactant proteins produced only by filamentous fungi [1]. They are small (≤20 kDa) secreted cysteine-rich proteins (SSCPs) that play pivotal roles in the fungal life cycle, helping with processes such as the formation of aerial structures by reducing the surface tension of the medium on which fungi grow, interactions with the surrounding environment, the adhesion of pathogenic fungi to plants, and the covering of spores to facilitate their dispersal in the air [2,3,4]. These unique proteins possess eight strictly conserved cysteine residues, forming four disulfide bridges to stabilize their tertiary protein structure [5]. HFBs can spontaneously self-assemble into an amphipathic monolayer at hydrophilic/hydrophobic interfaces that allows interactions between the fungi and their ecosystem [6,7].

Based on their hydropathy patterns and solubility characteristics, two classes of HFBs are described: class I and class II [8]. Class I hydrophobins (i) can be dissolved only by strong solvents, (ii) have been identified in Ascomycetes (class IA) and Basidiomycetes (class IB), (iii) form monolayers with rodlets (fibrillar amyloid-like substructures), and (iv) vary in their amino acid sequences [9,10]. In contrast, class II hydrophobins (i) can be dissociated in diluted organic solvents, (ii) are produced exclusively in Ascomycetes, (iii) are smaller than 10 kDa, and (iv) have higher conserved amino acid sequences than class I hydrophobins [11]. Recently, an intermediate class has been defined in *Aspergillus* and *Trichoderma* species [12,13].

Class II hydrophobins include cerato-ulmin [14], cryparin [15], and trihydrophobin [16]. Cerato-ulmin (CU) is a 7.6 KDa secreted hydrophobin toxin discovered from *Ophiostoma ulmi* and *Ophiostoma novo-ulmi*, the Dutch elm disease pathogens. It acts as a parasitic fitness factor that has been implicated in many aspects of development, including pathogenesis, adhesion, and the formation of reproductive structures [17,18,19]. Cryparin (CRP) is an abundant cell-surface-associated hydrophobin secreted by the chestnut blight fungus, *Cryphonectria parasitica*. It has lectin-like properties and binds to the cell wall of the fungus as well as being secreted into the media. CRP plays an essential role in the suitability of phytopathogenic fungi by facilitating the eruption of the fruiting bodies through the bark of the plant host [15,20]. Trihydrophobin (TH) is secreted from the ergot *Claviceps fusiformis*, which contains three domains of class II hydrophobins, each preceded by glycine/asparagine (GN)-rich regions [16]. Class II hydrophobins are usually between 80 and 125 amino acids in length, although they can be over 400 amino acids in length when including trihydrophobins [21].

In general, many SSCPs have been reported to function as fungal effectors [22]. Effectors are the most important class of proteins for interactions between a fungal pathogen and a plant host [23]. They enable the fungus to defeat PAMP-triggered immunity (PTI), a plant defense response that is raised by a pathogen-associated molecular pattern (PAMP). According to their localization inside the host plant, effectors are classified into apoplastic (cysteine-rich and secreted outside the host cell) and cytoplasmic (positively charged residues and secreted inside the host cell) [24,25]. Due to the similar properties between hydrophobins (especially those of class II) and effector proteins, many researchers have discussed the possible prominent role of class II hydrophobins in fungus–plant interactions [26,27]. Despite information on the function of hydrophobins for fungal pathogenesis, the role of these proteins in acting as plant defense elicitors and, further, the molecular mechanism of protein–ligand interactions remain unclear to date [19].

The elucidation of the tertiary protein structure is one of the key features for understanding biological processes at a molecular level, besides facilitating molecular docking studies [28]. The protein data bank (https://www.rcsb.org/, accessed on 7 July 2023) holds very limited structures under the keyword “hydrophobin class II”. For example, Ren et al. [29] reported the 3D structure of the class II hydrophobin NC2 (*Neurospora crassa* OR74A, PDB accession 4AOG) using the NMR method. In addition, Hakanpaa et al. [30] reported the 3D structure of the class II hydrophobin HFBII (*Trichoderma reesei*, PDB accession 2B97) using the X-ray diffraction method. The analysis and identification of the 3D structure of a certain protein using the X-ray crystallography or NMR spectroscopy methods are time-consuming and not successful with all proteins [31,32]. In silico bioinformatic approaches are an alternative tool developed to predict the 3D structure of proteins based on homology modeling using an unknown protein sequence [33]. The present study aimed to predict the functional domain and motif annotations of class II hydrophobins, characterize their physicochemical characteristics, explore high-template modeling for this group, study the conserved sites and evolutionary relationships of this family between fungal phytopathogens, and test their abilities to act as effectors using a variety of conventional computational tools.

## 2. Materials and Methods

### 2.1. Retrieval of Target Sequences

From the NCBI database, the amino acid sequences under the keywords “hydrophobin class II”, “cerato-ulmin”, and “cryprin” were filtered using HHfilter v3.3.0 (default parameters) to remove redundant proteins, and then the partial sequences and sequences related to non-pathogenic fungi were excluded. Finally, a total of 45 class II hydrophobin (HFBII) amino acid sequences of various fungal phytopathogen species were retrieved in the FASTA format from the NCBI database (https://www.ncbi.nlm.nih.gov/, accessed on 3 July 2023). The number of respective proteins with accession numbers and fungal sources is provided in Supplementary Table S1.

### 2.2. Analysis of Physicochemical Properties of the Proteins

The physicochemical parameters of the HFBII proteins were characterized using the ProtParam tool (http://web.expasy.org/protparam, accessed on 16 July 2023) of the ExPASy server [34]. The output data from this server included the molecular weight (MW), theoretical isoelectric point (PI), amino acid composition, atomic composition, estimated half-life, extinction coefficients (ECs), instability index (II), aliphatic index (AI), and grand average of hydropathicity (GRAVY). The hydropathy plot was analyzed and designed using the NovoPro server (https://novoprolabs.com/tools/protein-hydropathy, accessed on 17 July 2023).

### 2.3. Signal Peptide Prediction and Subcellular Localization Identification

Secreted proteins from the sequences that carry a signal peptide were predicted using SignalP 6.0 [35]. The DeepTMHMM V1.0.24 server was used to detect alpha and beta transmembrane proteins [36]. PredGPI was used to predict glycophosphatidylinositol (GPI) anchor motifs [37]. Anticipation of the subcellular localization and protein features was applied with the Bologna Unified Subcellular Component Annotator (BUSCA) server [38].

### 2.4. Modeling of 3D Protein Structures and its Evaluation

The 3D structures of all candidate HFBII proteins were designed by Alphafold2, trRosetta, and Multifold v1 [39,40,41]. The signal peptides were removed before homology modeling and a TM score > 0.50 was used as the threshold for reliably predicted folds [42]. The high pTM score models were verified and validated using Modfold v8.0 and the ProSA web server [43,44]. The Ramachandran plot was constructed using MolProbity and PDBsum [45,46]. The structural superpositions of the high-ranked predicted proteins and their experimental structures (PDB accession 4AOG) were performed using US-align [47]. All 3D structures and TM-align were visualized using UCSF Chimera 1.17.1 [48].

### 2.5. Functional and Structural Annotations of HBFII Proteins

The functional annotations were performed using InterPro 95.0 [49], Argot^2.5^ [50], and COFACTOR [51]. STRING v12 was used to determine the hydrophobin interactions with other related proteins, while Cytoscape v3.10 was used for the visualization of protein interactions [52,53]. EffectorP 3.0 and PHI-base were applied to search for HFBII effectors and virulence factors with their homologs in other pathogens [54,55]. The secondary structures were predicted using Quick2D (https://toolkit.tuebingen.mpg.de/tools/quick2d, accessed on 2 August 2023) with an e-value cut-off of 10^−3^, the UniRef90 database was used for MSA generation, and the maximal No. of MSA generation steps was 3. We used 2dss for the visualization of the 2D structure results from the Quick2D output [56]. Disordered residues were predicted using the ODiNPred server with a cut-off of 0.5 [57]. Rupee was used for determining the structural similarity against SCOPe v2.08, CATH v4.3, and the PDB chain databases, downloaded on 16 July 2022 [58,59]. MEME suite 5.5.3 was used for motif discovery, with a maximum number of 15 motifs and an e-value of less than 0.05 [60].

### 2.6. Sequence Alignment and Evolutionary Analysis

The 45 HFBII amino acid sequences were aligned using the MUSCLE tool of the MEGA 11 software [61]. Alignment sequences were applied to detect conserved residues of the HBFII proteins, which were visualized using Jalview 2.11.2.7 [62]. In addition, the entropy plot for the detected conserved amino acid residues was estimated using the Sequence Database Entropy-one web server (https://www.hiv.lanl.gov/content/sequence/ENTROPY/entropy_one.html, accessed on 10 August 2023), where the cut-off for conserved residues was a Shannon’s entropy of <1 and a proportion of gap < 0.1. The phylogenetic tree was constructed with the MEGA 11 software using the maximum likelihood method and was displayed and visualized via iTOl V6 [63]. A selection pressure analysis was performed using HyPhy via the Datamonkey web server [64,65]. A branch-level test for episodic diversification selection was detected with aBSREL v2.3 by testing all branches [66]. A site-level test for pervasive purifying or diversifying selection was inferred with FUBAR v2.2 by testing all branches [67]. In addition, the Selecton server was used for the identification of site-specific diversifying and purifying selections [68]. The ConSurf web server with the default parameters was assigned for detecting the functional and conserved regions in selected proteins [69].

### 2.7. Active Site and Protein Docking Analysis

The active site of the selected HFBII was identified using the scfbio server (https://www.scfbio-iitd.res.in/dock/ActiveSite.jsp, accessed on 15 August 2023) and CASTp 3.0 [70]. A molecular docking analysis was performed using CB-Dock2 [71] between the selected HFBII receptor protein and ligand (chitin). This helped the study and predicted the role of the HFBII proteins as effector proteins against plant chitinases. The ligand was retrieved from the ZINC database (ZINC 24425833) (https://zinc.docking.org/, accessed on 25 July 2023) in sdf format. The active site locations were visualized using UCSF ChimeraX v1.6.1 [72].

## 3. Results and Discussion

### 3.1. Detection of Physicochemical Characters of HFBII Proteins

**Figure 1:**
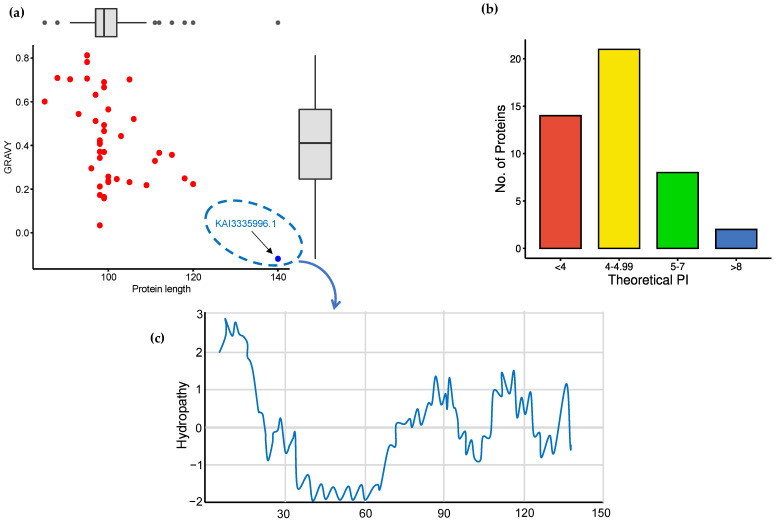
Physicochemical characteristics of the hydrophobin proteins: (**a**) protein length vs. GRAVY scores, where the negative values were categorized as globular (hydrophilic) proteins while the positive values were categorized as membrane (hydrophobic) proteins; (**b**) theoretical isoelectric point (PI) of hydrophobin proteins; and (**c**) hydropathy plot of *Ustulina deusta* cerato-ulmin HFBII.

From the NCBI database, 45 class II hydrophobin proteins were retrieved with dissimilar amino acid sequences. The output data of the physicochemical properties for these proteins, including the molecular weight, theoretical PI, instability index, aliphatic index, and GRAVY, were analyzed using the Expasy ProtParam tool (Table S2). Physical and chemical parameters can determine the behavior and stability of proteins under several in vitro conditions [73]. In this study, the length of the hydrophobin proteins ranged from 85 to 140 amino acids, but the majority were around 100 amino acids (Figure 1a). Moreover, the molecular weight (MW) ranged from 8.6 kDa to 13.46 kDa with an average of 10 kDa, which agreed with several works [9,30]. For the theoretical PI values, most proteins (77.7%) tended to be acidic below a PI of 5.0 (Figure 1b), where the theoretical PI of a protein is the pH at which the net charge carried by its surface equals zero [74]. Only two proteins (XP_003002035.1 and AAY89101.1) belonging to the genus *Verticillium* (*V. alfalfa* and *V. dahlia*, respectively) tended to be alkaline, with a PI of about 0.8, showing different features than the other HFBII proteins. Sixteen (35.3%) proteins were considered unstable according to their instability index (II), with cut-off values of <40 and >40 (Table S2). The instability index (II) of proteins lower than 40 was predicted to be stable [75]. The aliphatic index (AI) is an indicator of the thermal stability of proteins: an increase in the AI increases the stability of proteins at high temperatures [76]. The AI values of the studied proteins reflected the high thermostability of most hydrophobin proteins (53.93–110.95) over wide temperature ranges (Table S2). GRAVY is one of the important parameters studied that determines the hydrophilic or hydrophobic nature of proteins [77]. All proteins showed positive GRAVY scores except one protein (KAI3335996.1), which showed a negative GRAVY score (−0.119) (Figure 1a). The positive GRAVY score values indicated the hydrophobicity of the proteins, while the negative score value indicated hydrophilicity. Xu et al. [78] reported similar results about PI values, but we disagreed about the GRAVY score, where all the proteins of their work were hydrophobic, with GRAVY scores ranging from 0.333 to 0.967. In addition, we noticed that 41 proteins contained eight cysteine residues as described for the hydrophobin family, while only 4 proteins contained nine cysteine residues (Table S2).

### 3.2. Signal Peptide Prediction and Subcellular Localization Identification

**Figure 2:**
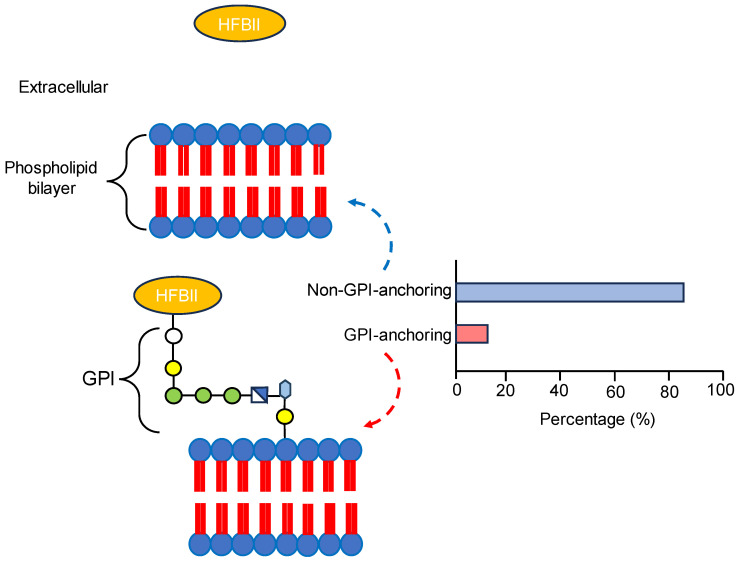
The number of GPI-anchored and non-GPI-anchored HFBII proteins with illustrated schematic diagram about GPI-anchoring localization outside the membrane.

Hydrophobin class II proteins were analyzed for the presence of signal peptides, transmembrane domains, and GPI anchors as described in the Section 2. The results showed that all the proteins carried signal peptides, but there was no evidence for the presence of alpha helices or beta proteins across the membrane. Huang et al. [79] and Neuhof et al. [80] also reported that there is a signal peptidase in the N-terminal region of HFBII proteins without a transmembrane helix. Out of 45 HBFII proteins, only 6 (13.3%) proteins were attached to the membrane by a GPI anchor (Figure 2, Table S3). The six proteins were AAB41284.1, KAB2579811.1, KAH8763703.1, KKY33170.1, KUI69349.1, and XP_047765241.1. The presence of GPI-anchored HFBII proteins is considered exclusive data about this family. GPI anchoring is a post-translational modification in the ER of eukaryotes, including fungi, and is important for development and pathogenicity [81]. Chun et al. [82] reported that the GPI-anchoring proteins of *Cryphonectria parasitica* are essential for virulence and phytotoxicity through an antioxidant barrier against host defenses, are active phytotoxic factors for pathogenicity, and are antiviral factors. In addition, Timmermans et al. [83] demonstrated the involvement of GPI-anchored proteins in cell wall remodeling, virulence, and the adhesion function of *Candida glabrata* to host cells. According to the previous information, the subcellular localization prediction of all proteins is termed “extracellular space” (GO:0005576).

### 3.3. Modeling of 3D Protein Structures and Model Evaluation

**Figure 3:**
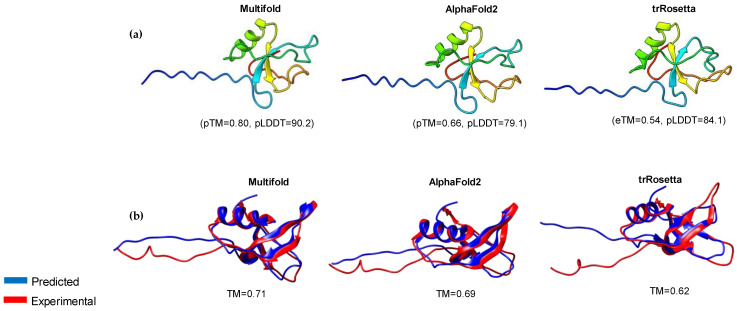
Homology modeling of representative HFBII protein: (**a**) three-dimensional models of *Verticillium dahlia* protein (XP_009650899.1) were generated using MultiFold, AlphaFold2, and trRosetta, showing TM-scores and pLDDT values; (**b**) structural superposition between the experimental (PDB: 4AOG) and predicted structures for the selected HFBII protein.

**Figure 4:**
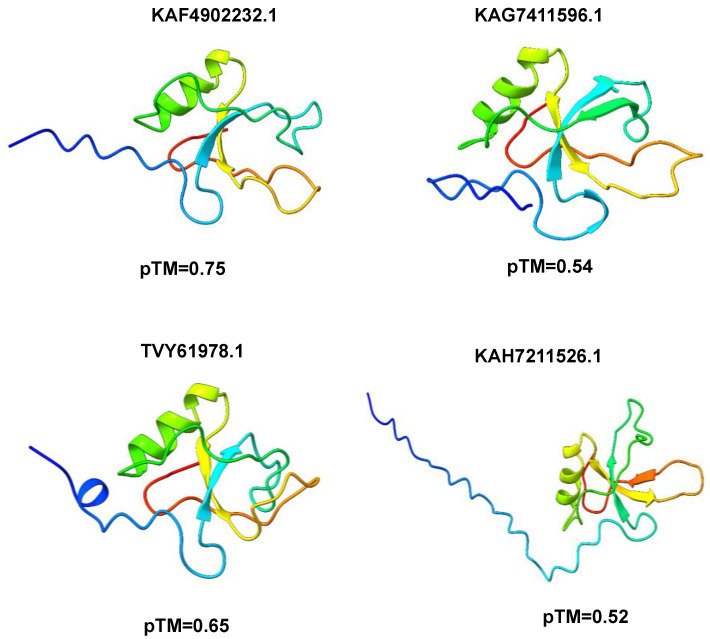
Three-dimensional (3D) models of other representative fungal proteins that resemble HFBII proteins, with different pTM scores in Multifold v1.

**Figure 5:**
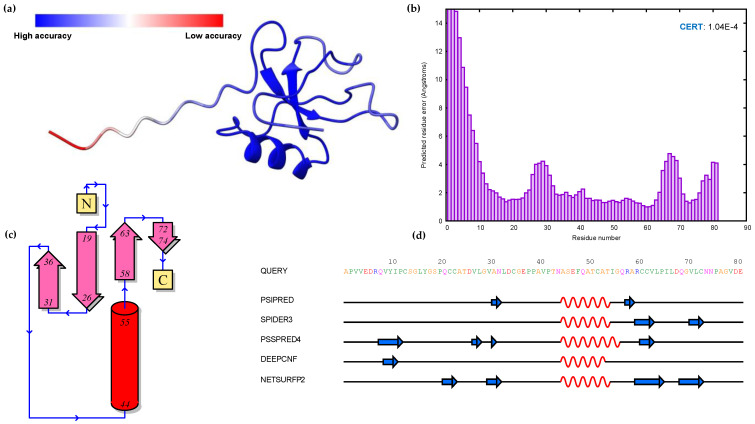
Model validation of protein (XP_009650899.1) and two-dimensional structure prediction: (**a**) B-factor coloring, indicating the protein residue quality; (**b**) protein model evaluation using ModFOLD8, representing the confidence and *p*-value; (**c**) schematic and topology diagram showing the secondary structural elements in the protein; and (**d**) comparative method, including five tools for predicting the 2D structure of HFBII proteins using the Quick2D server and visualization with 2dSS.

The prediction of the 3D structures of HFBII proteins is crucial due to the limited experimental data and their paucity in scientific papers. The structures of these proteins were predicted using different computational servers (Alphafold2, trRosseta, and Multifold) and the predicted models were superposed against the experimental ones with an accepted TM-align score > 0.5 (Figure 3a,b) [84]. Multifold v1 showed higher modeling precision than the other tools, at the level of both pTM and pLDDT (Figure 3 and Figure 4). The HFBII protein with the accession number “XP_009650899.1” gave the highest values in comparison to the other proteins, with the confidence and *p*-value “CERT: 1.04 × 10^−4^” according to the ModFold8 server (Figure 5a,b). The accuracy of the HFBII protein model was measured using a Ramachandran plot [85] and the result (97.5%) was satisfactory (Figure S1). The PROSA web server was used to analyze the protein structure by matching the predicted with the experimental structures using the statistics of the Cα of the mean force to evaluate the quality of the predicted proteins [86]. The output Z-score plots from the PROSA server revealed that the predicted protein models were within the range of the experimentally determined structures using the NMR method (Figure S2).

### 3.4. Functional and Structural Annotations of HBFII Proteins

**Figure 6:**
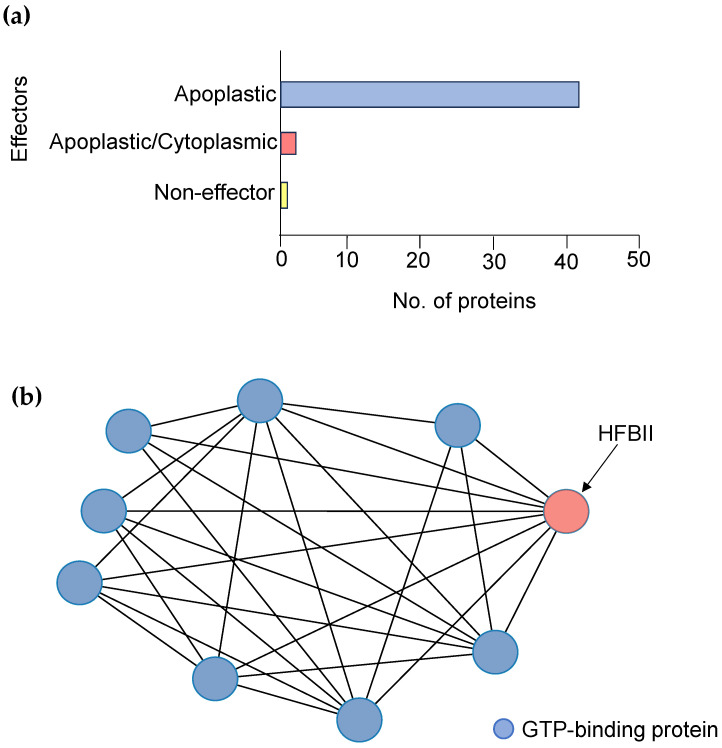
(**a**) Bar graph illustrating the effector and non-effector HFBII proteins; (**b**) STRING PPI network analysis between representative query HFBII (XP_009650899.1) and GTP-binding proteins. The average node degree is 5.6, the average local clustering coefficient is 0.778, and the PPI enrichment *p*-value is 5.28 × 10^−5^.

InterPro 95.0 and Argot^2.5^ were used for the functional annotation of the studied HFBII proteins according to the sequences, while the COFACTOR tool predicted the functional annotation of the proteins according to their structures [87]. The most annotated GO terms based on the biological processes were termed “pathogenesis” (GO:0009405), while the extracellular region (GO:0005615) encountered the dominant GO term (cellular component) for all proteins. There are no data about the molecular function of these proteins that could be detected by the annotation tools. The pathogenesis GO term indicates the role of these proteins in inducing an abnormal state inside their hosts [88]. The prediction of effectors among the HFBII proteins of phytopathogens is an essential criterion, although their prediction is a challenging task [89]. Therefore, we used EffectorP 3.0, a machine learning program, to construct the model depending on a variety of amino acid features [90]. From the forty-five HFBII proteins, forty-two proteins were classified as apoplastic effectors and two putative proteins were classified as apoplastic/cytoplasmic effectors that belonged to *Verticillium* spp., while one protein (CDK12896.1, *Geosmithia langdonii*) was found with no effector prediction (Figure 6a, Table S4). PHI-based data were used to compare the putative effectors with virulence genes that showed homology with other phytopathogens and classify the proteins into different categories [91,92]. According to the PHI annotation, all the effector proteins were categorized as having a reduced virulence that was encoded by the Fghyd5 [3] (PHI:9245) of *Fusarium graminearum* with different scores (Table S5), which helps the fungal hyphae to penetrate through the water–air interface and likely helps conidia adhere to the plant host [3].

**Figure 7:**
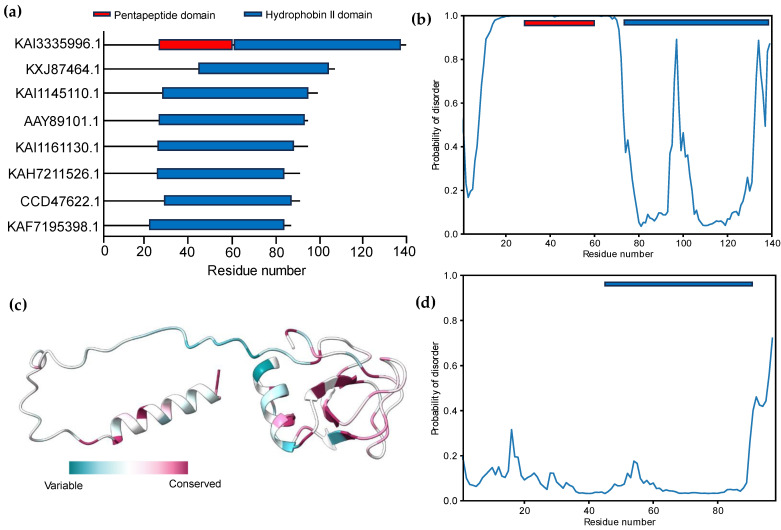
Domain and intrinsic disorder protein analysis: (**a**) domain profile of 8 selected HFBII proteins, illustrating a mutant bacterial domain in the KAI3335996.1 protein; (**b**) the prediction of the disordered regions for the hydrophobin II fusion protein with a pentapeptide domain; (**c**) conservation patterns for the KAI3335996.1 protein across several phytopathogen HFBII proteins that show a highly variable, disordered middle region (pentapeptide domain); and (**d**) the prediction of the disordered regions for the hydrophobin II representative protein without the pentapeptide fusion part.

The protein–protein interaction (PPI) between candidate effectors was analyzed using STRING v12.0. The results revealed that most effectors interacted with GTP-binding (GO:0005525) Rho proteins as a molecular function role (Figure 6b). Rho proteins regulate secretion and transcriptional activation, in addition to playing a role in cell transformation and signaling as effectors between cells [93]. The prediction of 2D structures for these proteins depended on the comparison between more than one tool using Quick2D. One alpha helix and two beta sheets were detected from the used tools (Figure 5c,d), and these findings matched with the 3D structure predictions. In addition, the 45 protein structures were assigned to SCOPe v2.08 and CATH v4.3 categories using RUPEE with a TM score cut-off of >0.5, but no aligned results were reported. The domain analysis of HFBII proteins ensured the presence of only one domain (hydrophobin II) in all the sequences stored in the InterPro database (IPR036686). Only one protein (KAI3335996.1, *Ustulina deusta*) possessed two domains: I) a pentapeptide repeats domain (IPR002989) from 30 to 60 residues and II) a hydrophobin II domain (Figure 7a). Pentapeptide repeats are found in many mycobacterial proteins involved in bacterial virulence [94]. This mutated region appeared more highly disordered than other proteins (Figure 7b–d) and the highly variable region (Figure 7c), so this region answered the question “why is this protein hydrophilic?” (Figure 1c).

### 3.5. Sequence Alignment and Evolutionary Analysis

**Figure 8:**
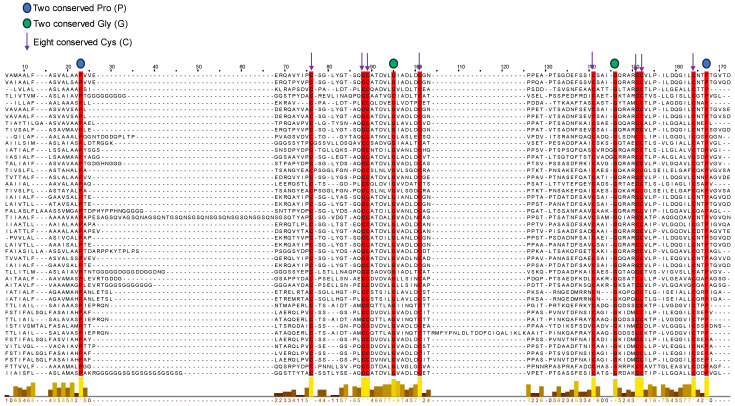
The conserved profile from alignment sequences of the selected HFBII proteins showed the twelve conserved residues (8 Cys, 2 Pro, and 2 Gly). The yellow color at the conservation bar below the figure indicates the 100% conservation residues.

The alignment of all the selected HFBII sequences was analyzed using the MUSCLE tool of the MEGA 11 program. From this alignment, a conserved pattern of amino acid residues was obtained for all the groups of protein sequences (Figure 8). The results of this profile illustrated four new conserved residues (two prolines and two glycines) other than the eight known conserved cysteines [95] of class I and II hydrophobin proteins (Figure 8). Shannon’s entropy in the residue analysis refers to the detection of the variation in characters in MSA [96], which also confirms the same conserved residue sites of the amino sequences (Figure S3). To better elucidate the evolutionary relationships among HFBII proteins, a phylogenetic tree and motif analysis were built based on the similarity of their amino acid sequences [97].

**Figure 9:**
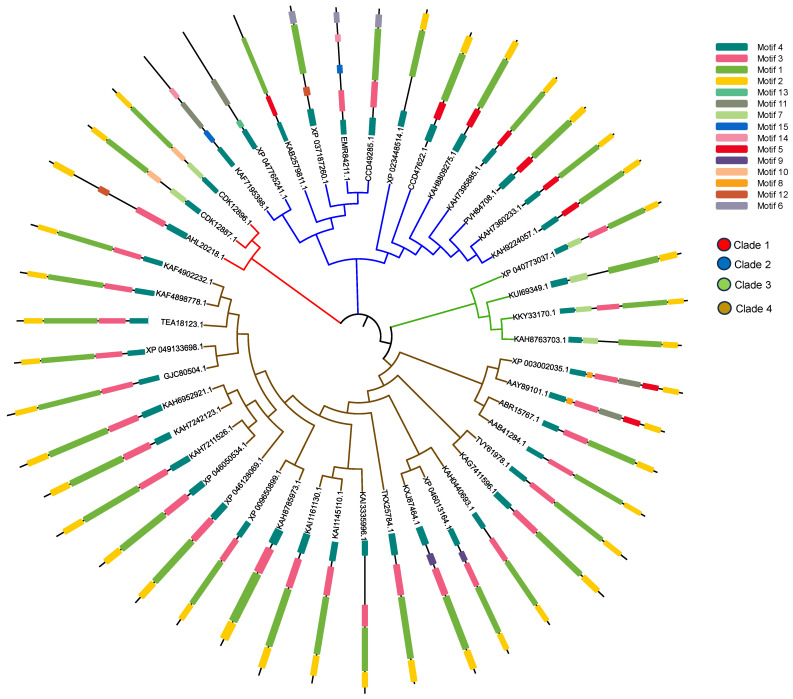
Construction of phylogenetic tree by MEGA 11 and visualization via iTol v6. Motif locations were identified using the MEME server.

According to the phylogeny analysis, the HFBII sequences were subdivided into four groups (clades), as shown in Figure 9. Moreover, among the four clades, group 4 had the largest number of HFBII members (25) with a high sequence and motif similarity. Group 3 was characterized by the presence of motif 7, while group 2 was characterized by the presence of motif 5. A further motif analysis showed that all the HFBII sequences shared motif 4 (signal peptide). Motif 1, motif 2, and motif 3 were present in most hydrophobin proteins and may have constituted the HFBII domain (Figure 9). Novel motifs were discovered between HFBII proteins such as motif 15 (KAF7195398.1 and EMR84211.1), motif 12 (XP_037187260.1 and AHL20218.1), motif 8 (specific to *Verticillium* species), motif 9 (specific to *Microdochium* species), motif 10 (specific to *Geosmithia* species), and motif 13, which were present only in *Fulvia fulva* (XP_047765241.1). All the motif symbols and the consensus are available and shown in Figure S4.

**Figure 10:**
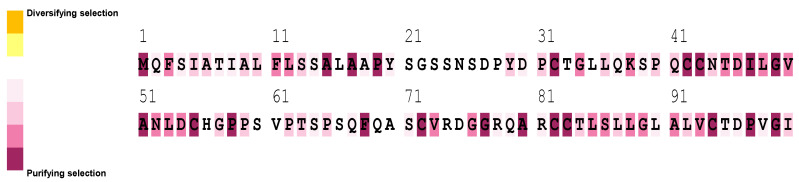
Positive selection analysis of the HFBII proteins using the Selecton server.

**Figure 11:**
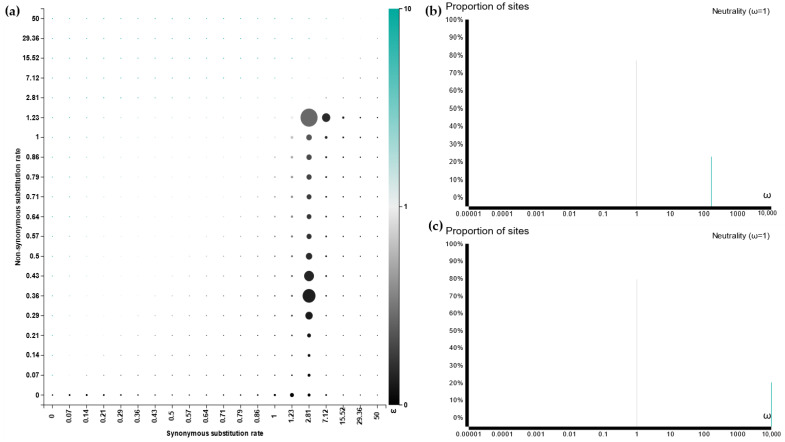
FUBAR and aBSREL evolutionary analyses: (**a**) FUBAR analysis of a coding sequence alignment to determine whether some sites have been subject to pervasive purifying or diversifying selection; (**b**) omega (ω) distribution over node 38 from the phylogenetic analysis using the aBSREL web server; and (**c**) omega (ω) distribution over a *Microdochium trichocladiopsis* node from the phylogenetic analysis using the aBSREL web server.

HFBII proteins have undergone an intricate process of evolution at the site level using the Selecton server and the FUBAR tool, while the branch level was analyzed using the aBSREL tool [98]. According to site-level evolution, there is no evidence for positive selection between the amino acid residues of the HFBII family (Figure 10 and Figure 11a). Based on a branch-level evolutionary analysis, an aBSREL discovered evidence of episodic diversifying selection on 2 out of 85 branches in the phylogeny analysis (Figure S5). A total of 85 branches were tested for diversifying selection. Significance was assessed using the likelihood ratio test (LRT) at a threshold of *p* ≤ 0.05, after correcting for multiple testing. The first branch (node 38) included AHL20218.1, CDK12887.1, and CDK12896.1 (Figure S5 and Figure 11a), which are represented as clade 1 on the phylogenetic tree (Figure 9). The first branch included only XP_046013164.1 (*Microdochium trichocladiopsis*), one of the group 4 members (Figure 11b).

### 3.6. Active Site and Protein Docking Analysis

**Figure 12:**
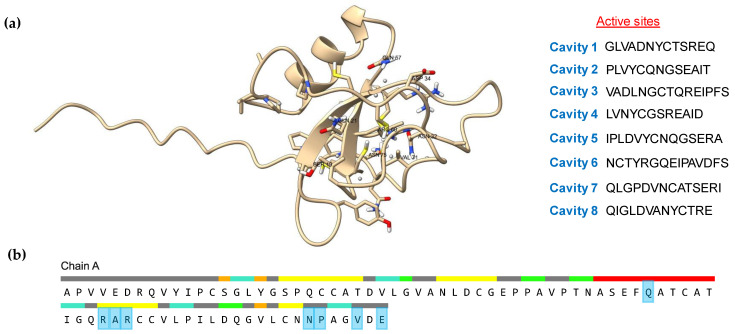
Active site information of an HFBII protein (XP_009650899.1): (**a**) eight cavities, detected by the scfbio server in the active site; (**b**) the amino acid residues (blue color) in the active site of the studied protein that were detected by the CASTp server.

**Figure 13:**
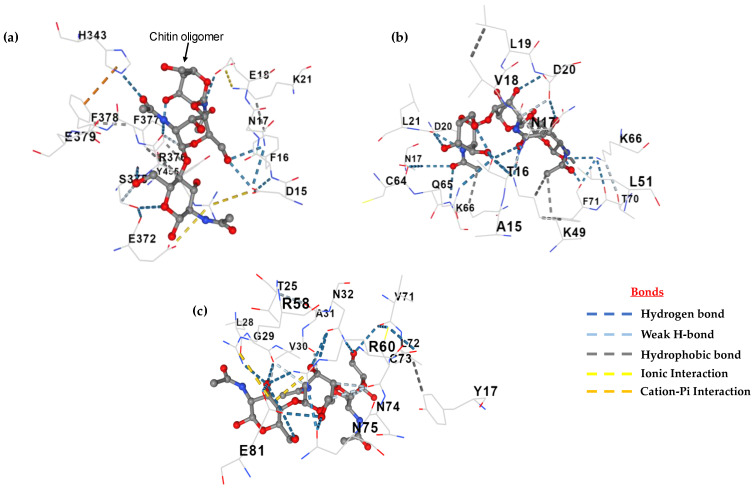
Molecular docking modeling between chitin oligomer (ligand) and (**a**) beta-N-acetylglucosaminidase (receptor); (**b**) experimental hydrophobin (receptor); and (**c**) predicted hydrophobin (receptor).

The active site of proteins is the surface region that facilitates binding with a specific substrate, which then undergoes catalysis [99]. The scfbio server demonstrated that eight cavities were present in the active site of the model protein (Figure 12a), while the CASTp server demonstrated eight amino acid residues (Figure 12b). As described previously, the most selected hydrophobins were predicted as apoplastic effectors by EffectorP 3.0. The widespread class of apoplastic effectors are chitin-oligomer-binding proteins that protect the fungal chitin layer from plant chitinases [100,101]. The predicted and experimental hydrophobins were evaluated for interactions against the chitin oligomer C_24_H_41_N_3_O_16_ (ZINC 24425833), while beta-N-acetylglucosaminidase (PDB 3wo8) was used as a control. The estimated free energy (ΔG) of binding between the beta-N-acetylglucosaminidase (control) and chitin was −7.8 Kcal/mol (Figure 13a) and the free energy (ΔG) between the experimental hydrophobin and chitin was −7.5 Kcal/mol (Figure 13b), while about −6.8 Kcal/mol was estimated between the predicted hydrophobin and chitin (Figure 13c). These results highlight the role of class II hydrophobins as apoplastic effectors. Frischmann et al. [102] and Baccelli et al. [103] reported that several cerato-platanin class-II-family hydrophobins were detected in the apoplast, but also remained bound to the chitin in the fungal cell wall and may have altered cell wall properties to protect the fungi from plant chitinases.

## 4. Conclusions

The class II (HFBII) hydrophobin family includes HFBII-domain-containing proteins that carry signal peptidase sequences. In this work, we retrieved and characterized HFBII proteins from 45 different phytopathogenic fungi. The evaluation of these proteins revealed that they were extracellular and acidic with a low molecular weight, a thermostable membrane (hydrophobic), and ranges of residues from 85 to 140. The MSA of the proteins ensured the presence of conserved proline (2) and glycine (2) plus the known cysteine (8), which provided rigidity and stability to the protein structure. The secondary structure analysis indicated the presence of one helix and two beta sheets located in the region of the HFBII domain. The functional annotation and the protein–protein interaction analysis illustrated that HFBII proteins may have protein-binding molecular functions (GTP-binding protein) and pathogenesis (GO:0009405), suggesting the possibility of their role as effectors, which was analyzed and predicted using molecular docking. The sequence and phylogenetic analysis confirmed the evolutionary conservation (site-level) of this member and discovered new motifs within the alignment sequences. The branch-level evolutionary analysis revealed the possibility of the episodic diversification of clade 1 from the other groups. The preliminary findings from this research will be useful in the future to encourage a deeper elucidation of this group’s mode of action and further provide a basis for exploring the function of HFBII in other processes.

## Data Availability

Not applicable.

## Supplementary Materials

The following supporting information can be downloaded at: https://www.mdpi.com/article/10.3390/microorganisms11112632/s1, Figure S1: MolProbity Ramachandran plots of the highest selected HFBII model (XP_009650899.1); Figure S2: Plot of residue energies with Z-scores of representative 3D HFBII protein models generated by PROSA web; Figure S3: Shannon entropy plot and sequence position in the multiple sequence alignment between 45 HFBII proteins for detecting the conserved residues; Figure S4: Fifteen motif symbols and consensus bits that were discovered using the MEME web server; Figure S5: The aBSREL method is a statistical framework that can be used to detect evidence of episodic diversifying selection in HFBII proteins; Table S1: Details of forty-five different HFBII proteins from different fungal sources used in this study; Table S2: All physical and chemical parameters of 45 selected HFBII proteins; Table S3: Detection of GPI-anchoring proteins for all candidate hydrophobin proteins by the PredGPI tool; Table S4: Effector proteins secreted from fungal phytopathogens that were detected by EffectorP 3.0; Table S5: Virulence factor genes with matched organisms against 45 class II hydrophobin proteins using the PHI database.
