# Supplementary material for: In Silico Evaluation, Phylogenetic Analysis, and Structural Modeling of the Class II Hydrophobin Family from Different Fungal Phytopathogens
Source: Microorganisms. 2023 Oct 26;11(11):2632. doi: 10.3390/microorganisms11112632 (PMC10672791; doi:10.3390/microorganisms11112632)
Supplement: Supplementary file 1 [file microorganisms-11-02632-s001.zip › Figure S1.pdf]

MolProbity Ramachandran analysis

rel_AF2MR_TS2_2_1_trimmed.pdb, model 1

General case

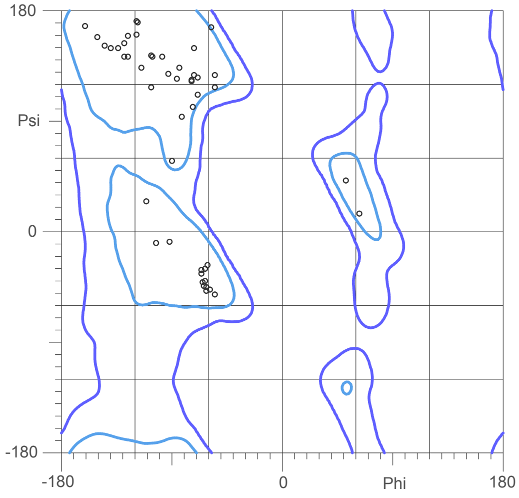

Isoleucine and valine

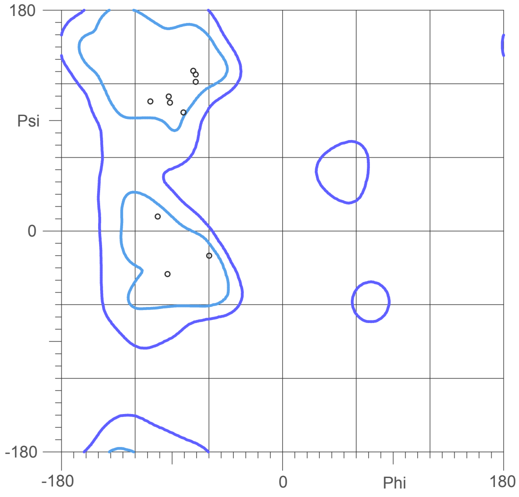

Pre-proline

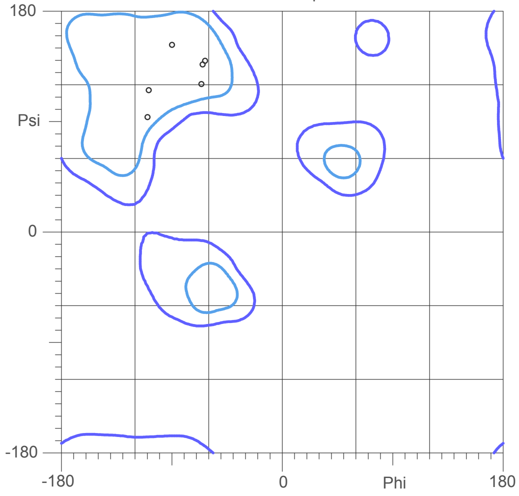

Glycine

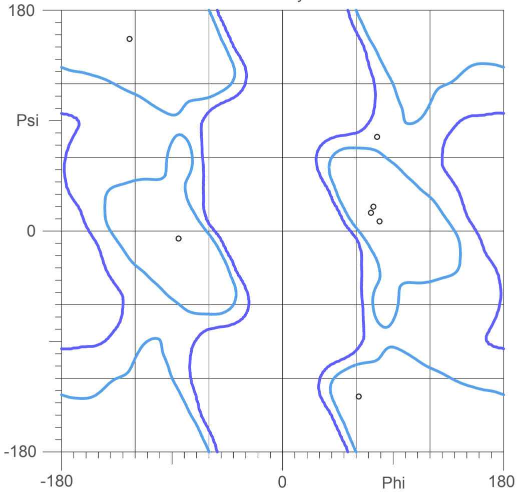

Trans proline

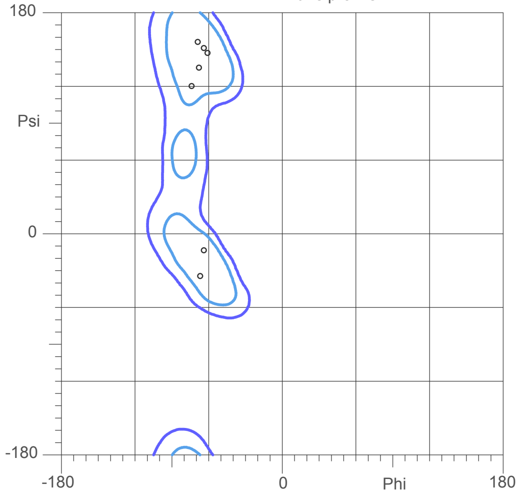

Cis proline

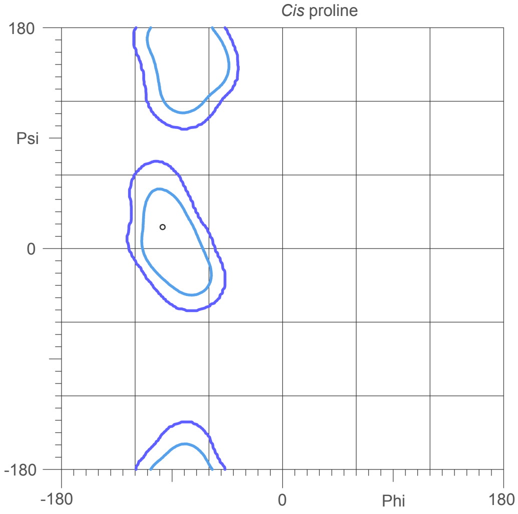

97.5% (77/79) of all residues were in favored (98%) regions.
100.0% (79/79) of all residues were in allowed (>99.8%) regions.

There were no outliers.

Lovell, Davis, et al. Proteins 50:437 (2003)
