# Supplementary material for: In Silico Evaluation, Phylogenetic Analysis, and Structural Modeling of the Class II Hydrophobin Family from Different Fungal Phytopathogens
Source: Microorganisms. 2023 Oct 26;11(11):2632. doi: 10.3390/microorganisms11112632 (PMC10672791; doi:10.3390/microorganisms11112632)
Supplement: Supplementary file 1 [file microorganisms-11-02632-s001.zip › Figure S2.pdf]

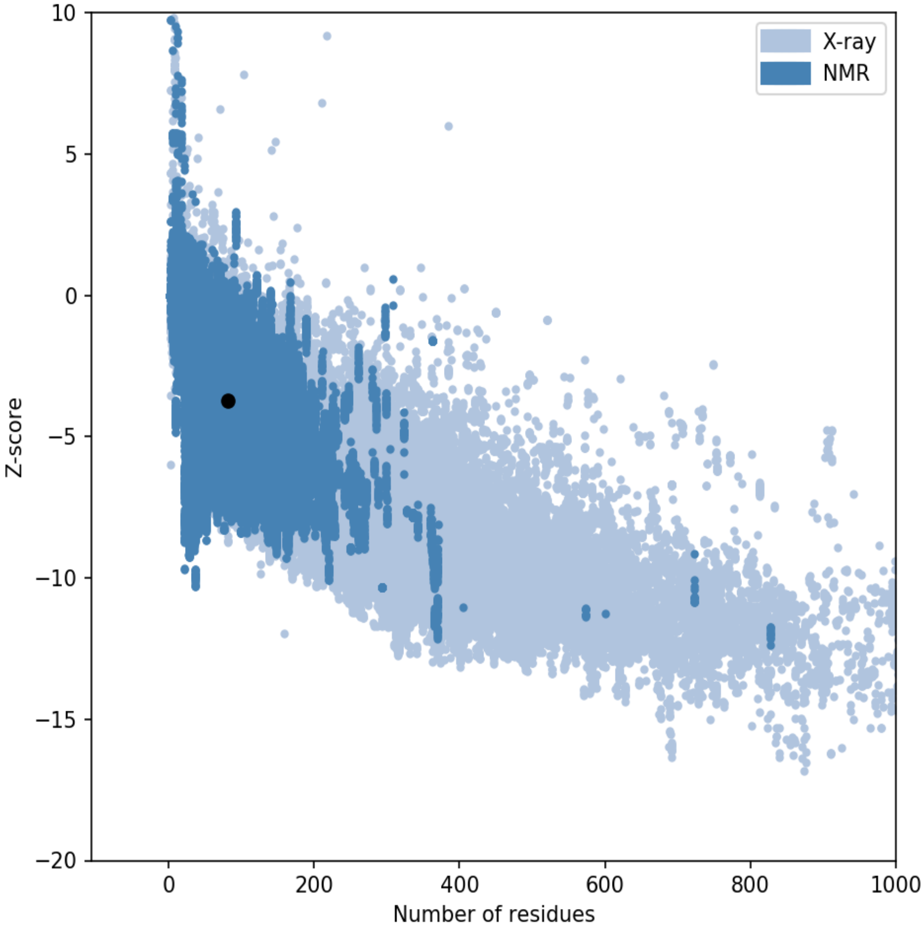

XP_009650899.1

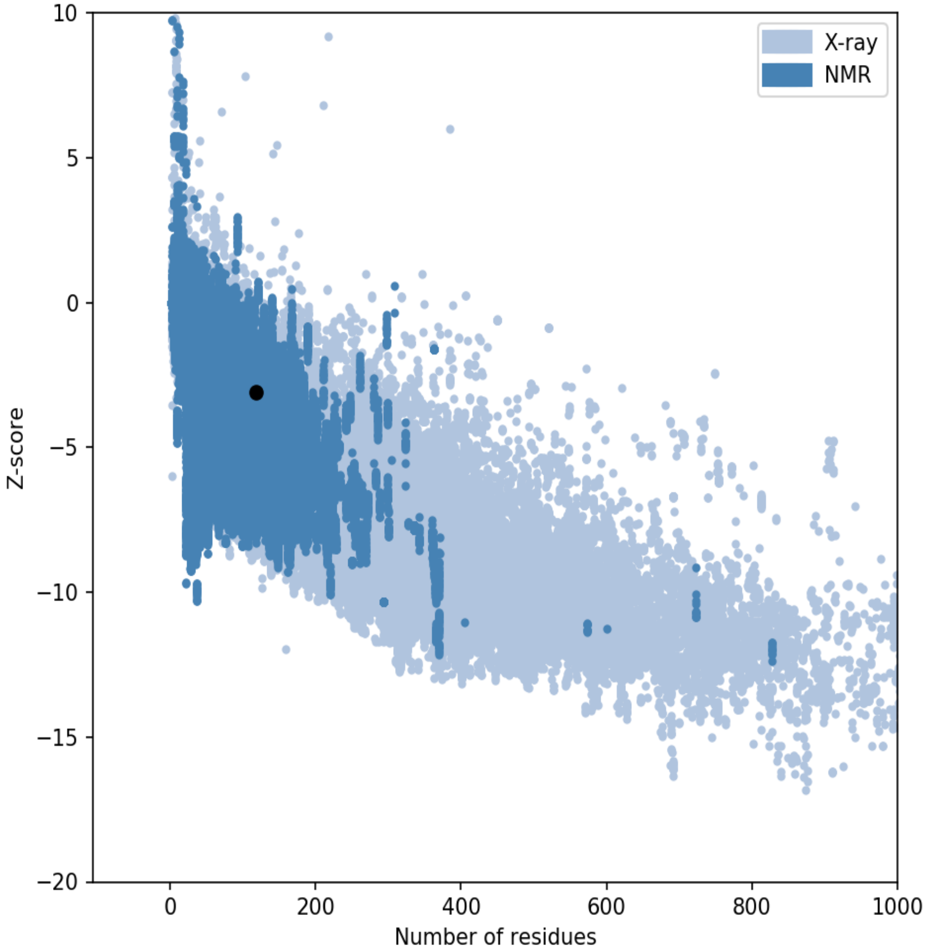

KAF4902232.1

Figure S2. Plot of residue energies with Z-scores of representative 3D HFB II protein models generated by PROSA web.
