# Supplementary material for: In Silico Evaluation, Phylogenetic Analysis, and Structural Modeling of the Class II Hydrophobin Family from Different Fungal Phytopathogens
Source: Microorganisms. 2023 Oct 26;11(11):2632. doi: 10.3390/microorganisms11112632 (PMC10672791; doi:10.3390/microorganisms11112632)
Supplement: Supplementary file 1 [file microorganisms-11-02632-s001.zip › Table S1.pdf]

Supplementary Table S1: Details of forty-five different HFBII from different fungal sources used in the study.

Serial No	Fungal Name	Protein name	Gene	Accession number	Sequence Length (aa)
1	*Botrytis cinerea BcDW1*	Hydrophobin class II	*N/A*	EMR84211.1	120
2	*Botrytis cinerea*	Hydrophobin class II	*Bhp2*	CCD47622.1	98
3	*Botrytis cinerea T4*	Hydrophobin class II	*Bhp3*	CCD49285.1	98
4	*Botrytis fragariae*	Hydrophobin class II	*N/A*	XP_037187260.1	98
5	*Cadophora sp. DSE1049*	Hydrophobin class II	*Bhp2*	PVH84708.1	95
6	*Cercospora beticola*	Cryparin	*N/A*	XP_023448514.1	102
7	*Clonostachys rosea*	Hydrophobin class II	*N/A*	AHL20218.1	98
8	*Colletotrichum camelliae*	cerato-ulmin	*N/A*	KAH0440693.1	103
9	*Colletotrichum fructicola*	Cryparin	*CRP*	KAF4898778.1	97
10	*Colletotrichum liriopes*	Cryparin	*N/A*	GJC80504.1	99
11	*Colletotrichum sidae*	Cryparin	*CRP*	TEA18123.1	98
12	*Colletotrichum spaethianum*	Cryparin	*N/A*	XP_049133698.1	99
13	*Colletotrichum viniferum*	Cryparin	*CRP*	KAF4902232.1	97
14	*Cryphonectria parasitica EP155*	Cryparin	*CRP*	XP_040773037.1	118
15	*Diaporthaceae* sp. PMI_573	Cryparin	*N/A*	KAH8763703.1	106
16	*Diaporthaceae* sp. PMI_573	cerato-ulmin	*CU*	KAH8785973.1	99
17	*Diaporthe ampelina*	cerato-ulmin	*N/A*	KKY33170.1	115
18	*Elsinoe australis*	cryparin	*N/A*	TKX25784.1	97
19	*Fulvia fulva*	cryparin	*N/A*	XP_047765241.1	88
20	*Fusarium avenaceum*	cerato-ulmin	*N/A*	KAH6952921.1	98
21	*Fusarium oxysporum*	cerato-ulmin	*N/A*	KAH7211526.1	98
22	*Fusarium oxysporum f. sp. cubense*	cerato-ulmin	*CU*	TVY61978.1	100
23	*Fusarium oxysporum f. sp. raphani*	cerato-ulmin	*CU*	KAG7411596.1	100
24	*Fusarium redolens*	cerato-ulmin	*N/A*	XP_046050534.1	98

25	*Fusarium solani*	cerato-ulmin	*N/A*	XP_046128069.1	96
26	*Fusarium tricinctum*	cerato-ulmin	*N/A*	KAH7242123.1	98
27	*Geosmithia langdonii*	Hydrophobin class II	*geo1*	CDK12896.1	105
28	*Geosmithia pallida*	Hydrophobin class II	*geo1*	CDK12887.1	112
29	*Lasiodiplodia theobromae*	cerato-ulmin	*CU_0*	KAB2579811.1	91
30	*Leotiomycetes* sp. MPI-SDFR-AT-0126	Hydrophobin class II	*Bhp2*	KAH7395885.1	95
31	*Leptodontidium* sp. 2 PMI_412	Hydrophobin class II	*Bhp2*	KAH9224057.1	95
32	*Microdochium bolleyi*	cerato-ulmin	*N/A*	KXJ87464.1	111
33	*Microdochium trichocladiopsis*	cerato-ulmin	*N/A*	XP_046013164.1	109
34	*Nemania diffusa*	cerato-ulmin	*N/A*	KAI1145110.1	100
35	*Nemania serpens*	cerato-ulmin	*N/A*	KAI1161130.1	99
36	*Ophiostoma quercus*	cerato-ulmin	*N/A*	ABR15767.1	99
37	*Ophiostoma ulmi*	cerato-ulmin	*CU*	AAB41284.1	100
38	*Pseudocercospora fuligena*	Cryparin	*N/A*	KAF7195398.1	85
39	*Rhexocercosporidium* sp. MPI-PUGE-AT-0058	Hydrophobin class II	*Bhp2*	KAH7360233.1	95
40	*Ustulina deusta*	cerato-ulmin	*N/A*	KAI3335996.1	140
41	*Valsa mali*	Cryparin	*N/A*	KUI69349.1	105
42	*Verticillium alfalfae VaMs.102*	Hydrophobin class II	*N/A*	XP_003002035.1	99
43	*Verticillium dahliae*	Hydrophobin class II	*VDH1*	AAY89101.1	99
44	*Verticillium dahliae VdLs.17*	cerato-ulmin	*N/A*	XP_009650899.1	98
45	*Xylogone sp. PMI_703*	Hydrophobin class II	*Bhp2*	KAH8809275.1	93
