# Supplementary figures and images for: In Silico Evaluation, Phylogenetic Analysis, and Structural Modeling of the Class II Hydrophobin Family from Different Fungal Phytopathogens
Source: Microorganisms. 2023 Oct 26;11(11):2632. doi: 10.3390/microorganisms11112632 (PMC10672791; doi:10.3390/microorganisms11112632)

Entropy

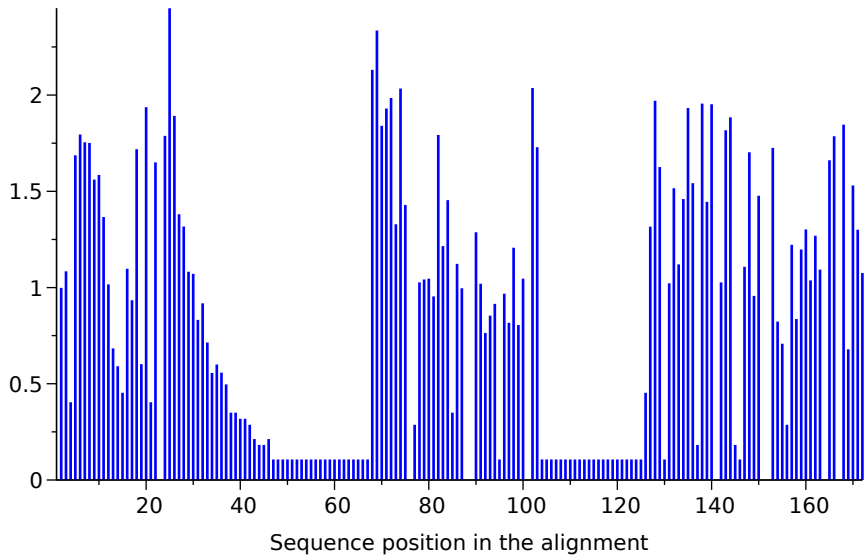

Supplement: Supplementary file 1 [file microorganisms-11-02632-s001.zip › Figure S3.pdf]

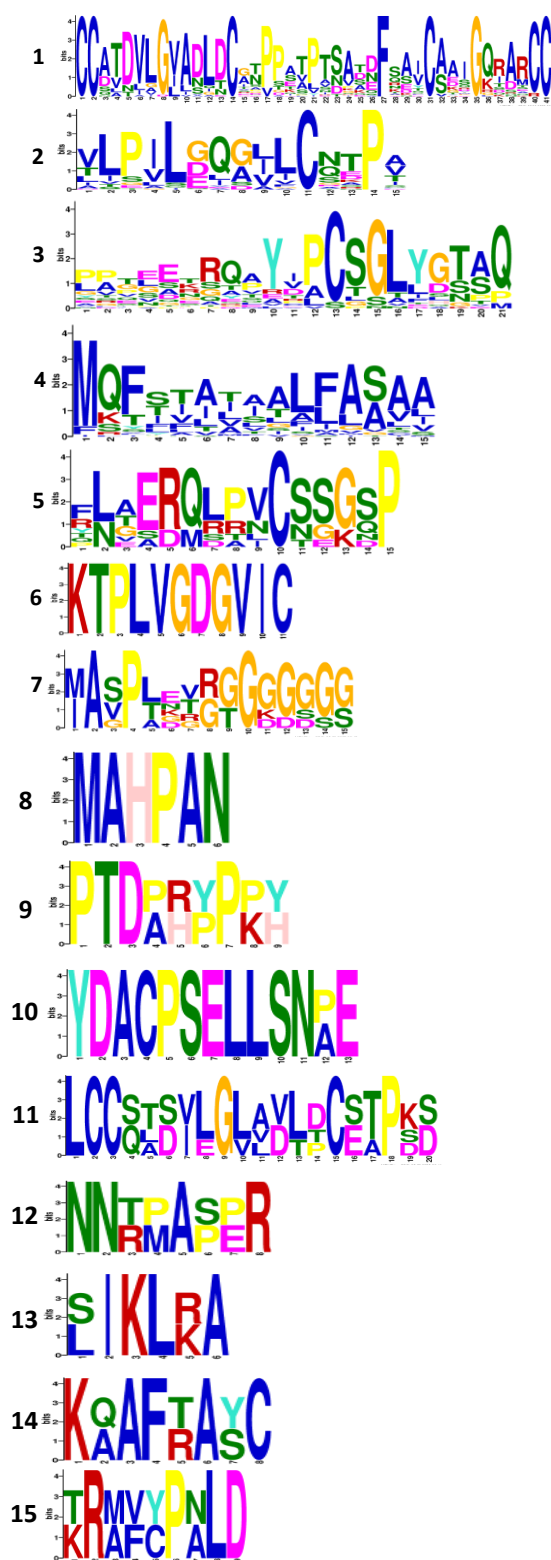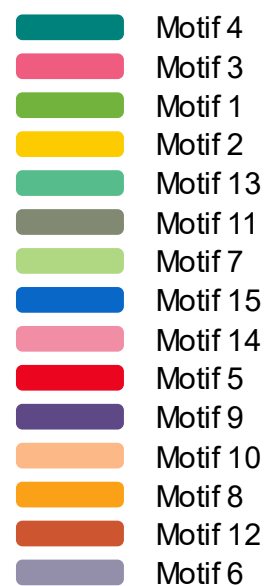

Supplement: Supplementary file 1 [file microorganisms-11-02632-s001.zip › Figure S4.pdf]

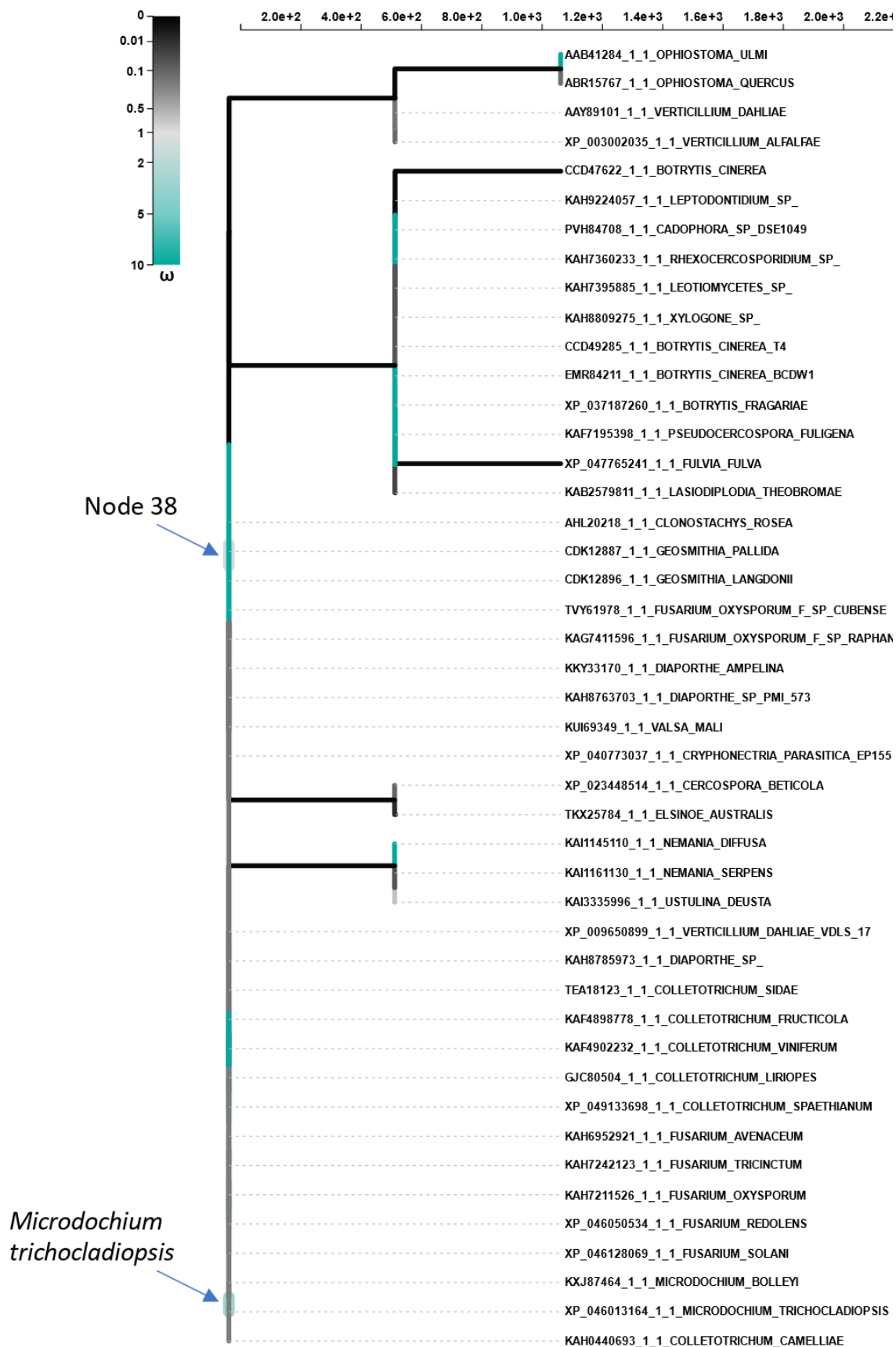

Supplement: Supplementary file 1 [file microorganisms-11-02632-s001.zip › Figure S5.pdf]
